# Reducing Health Anxiety in Patients With Inflammatory Bowel Disease Using Video Testimonials: Pilot Assessment of a Video Intervention

**DOI:** 10.2196/39945

**Published:** 2023-02-09

**Authors:** Joel Shor, Yusuke Miyatani, Etsuko Arita, Pohung Chen, Yusuke Ito, Hiroki Kayama, Jacob Reiter, Kaho Kobayashi, Taku Kobayashi

**Affiliations:** 1 Verily Life Sciences South San Francisco, CA United States; 2 Center for Advanced Inflammatory Bowel Disease Research and Treatment Kitasato University Kitasato Institute Hospital Tokyo Japan; 3 Medical Psychology Pharmaceutical Education Research Center, School of Pharmacy, Kitasato University Tokyo Japan; 4 Google Japan Tokyo Japan

**Keywords:** anxiety, bowel disease, chronic condition, chronic disease, chronic illness, Crohn disease, digital health, digital intervention, digital video testimonial, eHealth, experience, fear, gastroenterologist, gastroenterology, gastrointestinal, health anxiety, inflammatory bowel disease, internal medicine, Japan, life narrative, mental health, perception, perspective, pilot study, relaxation, stories, testimonial, ulcerative colitis, video

## Abstract

**Background:**

Health anxiety has many damaging effects on patients with chronic illness. Physicians are often unable to alleviate concerns related to living with a disease that has an impact on daily life, and unregulated websites can overrepresent extreme anxiety-inducing outcomes. Educational clinician video interventions have shown some success as an acute anxiolytic in health settings. However, little research has evaluated if peer-based video interventions would be a feasible alternative or improvement.

**Objective:**

This pilot study assesses the efficacy of anxiety reduction for patients with Crohn disease (CD) and those with ulcerative colitis (UC) by showing patient testimonial videos during hospital visits. It investigates the degree to which patient testimonials can affect state anxiety, and whether patients are comfortable enough with the technology to share their stories.

**Methods:**

Patients with CD (n=51) and those with UC (n=49) were shown testimonial videos of patients with CD during their physician consultations at Kitasato University Kitasato Institute Hospital in Japan. The video testimonials were collected from Dipex Japan, the Japan branch of an international organization specializing in understanding patient experiences. Patients completed a Visual Analogue Scale for Anxiety before and after viewing the videos, a Hospital Anxiety and Depression Scale (HADS) survey before the videos, and satisfaction surveys. Patients receiving infusion therapy participated in the study while receiving treatment to minimize hospital workflow disruption.

**Results:**

Anxiety reduction, on the Visual Analog Scale for Anxiety, was significant in the entire cohort both when viewed as an ordinal variable (*P*=.003, *t*_98_=1086.5) and as a continuous variable (*P*=.01, *t*_94_=–2.54, 90% CI –3.47 to –0.72). Eighty percent (n=15) of patients with high HADS Anxiety (HADS-A) scores and 71% (n=24) of patients with high starting state anxiety experienced reduced anxiety after watching testimonials. Patients with high state anxiety but low HADS-A scores experienced anxiety reduction (69%, n=16). Forty-two percent (n=100) of patients responded that they would share their stories for future users. When patients with UC received testimonials from patients with CD, 71% (n=49) of patients reported that they were relevant despite differences in condition.

**Conclusions:**

Our pilot results suggest that patient testimonial videos can reduce illness-related state anxiety for patients with CD and those with UC, especially in those with higher baseline state anxiety. The success of this study in reducing anxiety and achieving patient involvement suggests that video interventions for reducing anxiety might be a low-cost intervention that could scale to any number of hospitals, suggesting that technology can help scale up efforts to record and share patient testimonials. Future work can establish whether patient testimonials can be helpful in other contexts, such as before major surgeries or when a family member receives a difficult diagnosis.

## Introduction

### Background

Managing health anxiety is essential in treating chronic illness [1,2]. Physicians can provide patients with facts about treatment based on evidence but are often unable to address anxieties related to the experience of living with a chronic illness. Websites can be a useful tool for patients to share experiences, but they can also increase patients’ concerns about their treatment and hamper medication adherence [3,4].

These challenges are amplified in patients with inflammatory bowel disease (IBD), who are estimated to have a 2- to 6-fold increased odds for developing an anxiety disorder [5-7]. IBD and anxiety disorders have a bidirectional relationship, highlighting the importance of managing anxiety disorders in the treatment of IBD [6,8].

Recent literature has examined how clinician educational video interventions can be used as an acute anxiolytic in health settings [9-12]. Several randomized trials have shown success in a wide variety of conditions and disorders [13-15], including IBD [16]. However, results have not always been promising in IBD [17-19]. Further literature has identified the anxiolytic effect when IBD education is delivered not by a clinician, but from peers with IBD who have shared experiences to the patient [20]. However, peer-based education faces challenges of scale and are, therefore, prime candidates for digital innovation. Yet, only one publication to date has evaluated if peer-based education effects translate over video in hospital settings [21], and, to our knowledge, there have been no publications to date investigating peer-based interventions by video for specifically IBD.

### Objectives

In this work, we describe a pilot study that assessed the efficacy of anxiety reduction for patients with Crohn disease (CD) and those with ulcerative colitis (UC) by showing patient testimonial videos during hospital visits. This pilot study investigates whether patient testimonials can be integrated into the normal hospital workflow, and whether patients are comfortable enough with the technology to share their stories.

## Methods

### Site and Participants

The study was conducted at the outpatient clinic of the Center for Advanced IBD Research and Treatment at Kitasato University Kitasato Institute Hospital. Randomly selected patients with a confirmed diagnosis of CD or UC were included in the study if they were older than 20 years. Patients were excluded from the study if they did not understand Japanese or participated in any other clinical trials. A total of 100 patients were included. See Table 1 for patient descriptives.

**Table 1:** Descriptives of patients and self-report measures (N=100).

Characteristic			Value
**Condition, n**			
	UC^a^		49
	CD^b^		51
**Sex, n**			
	Male		63
	Female		37
Age (years), median (IQR)			41.5 (30-50)
Years since diagnosis, median (IQR)			10 (5-20)
**Disease severity^c^, median (IQR)**			
	CD		59 (37.5-108.5)
	UC		0 (0-1)
**Hospital Anxiety and Depression Scale–Anxiety score, mean (SD)**			
	All		5.6 (4.3)
	CD		5.0 (4.4)
	UC		6.3 (4.3)
**Starting Visual Analog Scale for Anxiety score, mean (SD)**			
	All		26.8 (25.1)
	CD		28.3 (26.4)
	UC		25.2 (23.7)
**Patients per anxiety category^d^, n**			
	Low HADS-A^e^, Low baseline VAS-A^f^		64
	Low HADS-A, High baseline VAS-A		16
	High HADS-A, Low baseline VAS-A		8
	High HADS-A, High baseline VAS-A		7
**Satisfaction surveys^g^, n**			
	**Willing to share story**		
		Yes	43
		No	48
		No response	9
	**How would you be willing to share your story? (Multiple responses allowed)**		
		Video	18
		Document	23
		Audio only	7
	**Were the testimonial videos relevant to you?**		
		Yes^h^	71
		No^i^	29
	**Were the testimonial videos helpful?**		
		Yes^j^	54
		No^k^	8
		Unsure^l^	38

^a^UC: ulcerative colitis.

^b^CD: Crohn disease.

^c^Crohn’s Disease Activity Index for CD [22] and Partial Mayo Score for UC [23].

^d^Low HADS-A is below 11 [24]. Low VAS-A is below 47 [25].

^e^Hospital Anxiety and Depression Scale

^f^Visual Analogue Scale for Anxiety

^g^Questions were presented to patients in Japanese; English translations shown in table above.

^h^Among patients with UC, the *yes* response was provided by 35 participants.

^i^Among patients with UC, the *no* response was provided by 14 participants.

^j^Among patients with UC, the *yes* response was provided by 28 participants.

^k^Among patients with UC, the *no* response was provided by 5 participants.

^l^Among patients with UC, the *unsure* response was provided by 16 participants.

### Ethics Considerations

This study was approved by the Research Ethics Committee of Kitasato University Kitasato Institute Hospital (#21006). Data analyzed in this study were anonymized. Patients signed consent waivers in Japanese consistent with Kitasato University Kitasato Institute Hospital practices and were not compensated.

### Anxiety

#### Visual Analogue Scale for Anxiety

The primary outcome metric for this study was the Visual Analogue Scale for Anxiety (VAS-A) [25], which measures anxiety in the moment (state anxiety). All patients completed the VAS-A before and after the video intervention. Patients self-reported their level of anxiety on a printed line, with scores calculated as the distance in millimeters. Scores were normalized between 0 and 100 [26].

#### Hospital Anxiety and Depression Scale

All patients completed the Hospital Anxiety and Depression Scale (HADS) survey [24]. Seven items comprise the HADS Anxiety (HADS-A) subscale. For our population, we used the clinically validated Japanese translation of the HADS [27]. Since HADS assesses symptoms over the previous week, HADS was not readministered after video intervention.

### Testimonial Videos

The videos were collected from DIPEx Japan [28] using the “Health and illness narrative” methodology [29]. Narratives from patients with CD that are publicly available are collected and used with explicit consent from DIPEx Japan. The same 6 patient testimonial videos were selected as “required viewing” (12 minutes) for everyone in the pilot, and 22 videos were included as “optional viewing.” These videos were selected to be diverse in subject matter, tone, and speaker characteristics (Multimedia Appendix 1).

### Hospital Senpai Video Platform

**Figure 1:**
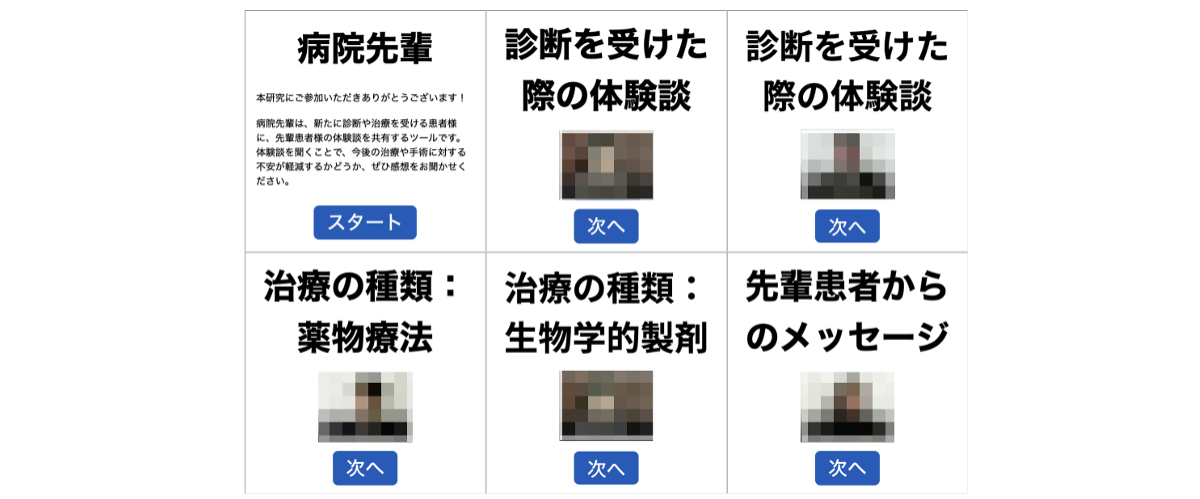
A screen capture of 6 pages of the Hospital Senpai video viewing software. Patient images are blurred in this screenshot but not the actual software.

Patients met with physicians before participating in the Hospital Senpai pilot. Participants completed the VAS-A and HADS, and then engaged with the testimonial videos through the software (Figure 1). Patients watched a linear progression of required videos, optionally viewed additional videos, then completed postvideo VAS-A and satisfaction surveys. Patients who received infusion therapy watched videos and completed the surveys during their infusion treatment.

### Satisfaction Surveys

Patients completed an 11-question multiple choice and free-form survey on usability and satisfaction at the end of the study. Users answered questions in Japanese such as whether the content was relevant, how easy the video viewing process was, and whether the patients would share their story for future users.

### Statistical Analysis

We compare the VAS-A scores before and after video testimonials. In some domains, the VAS values are not normally distributed [30] and only the order of values matters [31] so we report both a paired, 2-sided Student *t* test and a paired, 2-sided Wilcoxon signed rank test. The Wilcoxon signed rank test is a distribution-free alternative to the paired Student *t* test. Patients who did not complete part of the survey or questionnaire were excluded from the relevant analyses. Following the previous work, we exclude from the *t* tests outliers more than 2.5 SD from the mean [32] (Multimedia Appendix 1). We use correlation and partial correlation [33] to explore the relationship between variables.

## Results

### Anxiety Reduction

**Figure 2:**
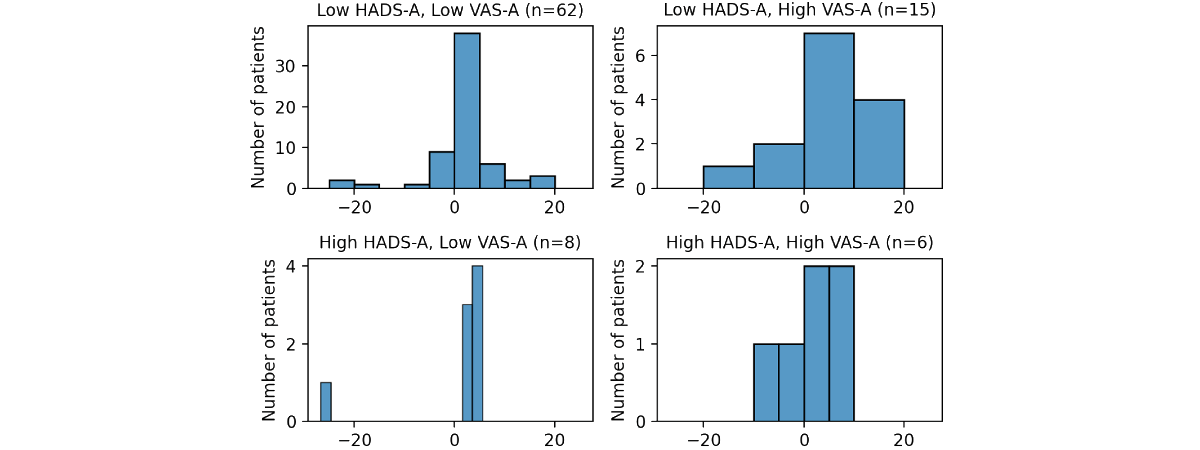
Histogram plots of prevideo Visual Analogue Scale for Anxiety (VAS-A) scores minus postvideo VAS-A scores for different baseline VAS-A and HADS Anxiety (HADS-A) cohorts. Positive scores mean reduced anxiety. Patients with incomplete Hospital Anxiety and Depression Scale (HADS) responses were dropped.

There was a statistically significant decrease in state anxiety (VAS-A) before and after watching testimonial videos across all patients both when interpreting VAS-A as an ordinal variable (*P*=.003, *t*_98_=1086.5) and a continuous variable (*P*=.01, *t*_94_=–2.54, 90% CI –3.47 to –0.72). The largest magnitude of anxiety reduction occurred in patients with high starting VAS-A scores (90% CI –7.89 to –1.86, n=22), but the most consistent reduction effect was seen in 80% of patients (n=15) with high HADS-A (see Figure 2).

**Figure 3:**
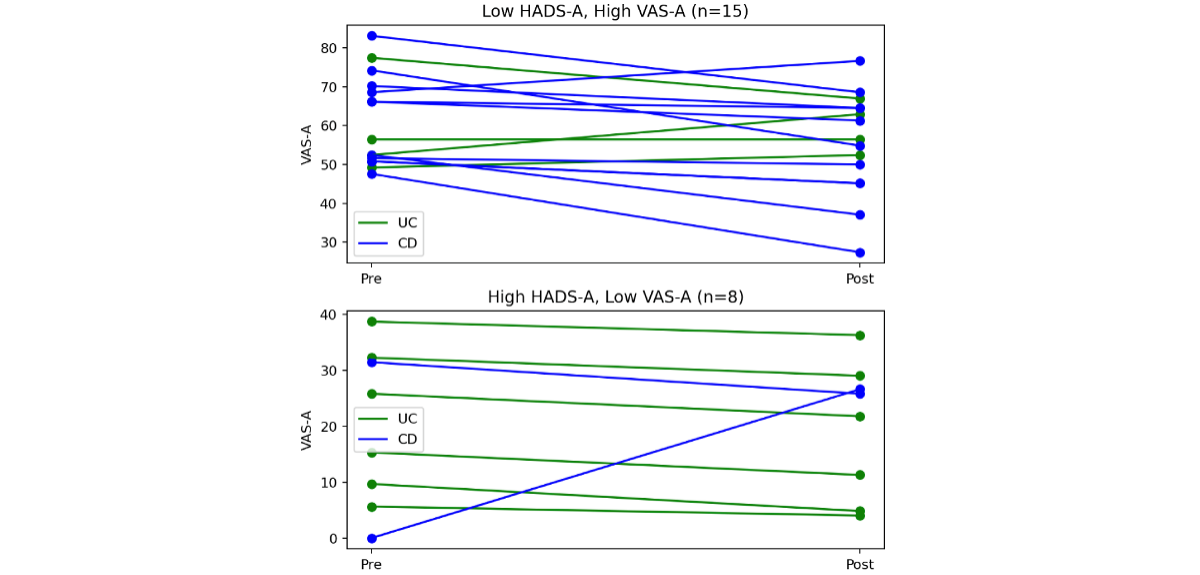
Pre–Visual Analogue Scale for Anxiety (Pre-VAS-A) and Post-VAS-A scores for 2 subgroups. CD: Crohn disease; UC: ulcerative colitis.

Sixty-nine percent (n=16) of patients with low HADS-A but high baseline VAS-A experienced a reduction in anxiety (see Figure 3). Baseline VAS-A anxiety and HADS-A scores are correlated both as continuous and ordinal variables (*r*=0.41, *ρ*=0.40), and HADS-A was primarily correlated with anxiety reduction through baseline VAS-A (*r*=0.12 between HADS-A and anxiety reduction, and 0.014 partial correlation when controlling for baseline VAS-A), indicating that video testimonials can be effective for patients without anxiety on a longer timescale.

### Patient App Usability, Satisfaction, and Participation

Among all patients, 71% (n=100) responded that the videos were relevant and only 8% (n=100) said that they were not helpful. Importantly, patients with UC responded similarly even though all testimonial videos were from patients with CD (71% relevant, 10% not helpful, n=49). In addition, 42% (n=100) of all patients responded that they would be willing to share their stories on the platform for future patients.

## Discussion

This study suggests that patient testimonial videos can reduce state anxiety during hospital visits for patients diagnosed with IBD. It also demonstrates that video testimonials can be effectively integrated into the normal hospital workflow. Finally, surveys show that patients are willing to share their stories for future patients as part of the program.

Patients with high starting state anxieties demonstrated the greatest reduction in state anxiety. Similarly, patients with high starting state anxieties and low HADS-A consistently experienced reduced anxiety, indicating that patients without a formal anxiety disorder could potentially benefit from video testimonial intervention. For reference, the psychopharmacological intervention study with closest methodology investigating acute anxiolytic effect in the range of minutes-to-hours found a comparable VAS-A reduction [34]. Additionally, a large fraction of patients were willing to share their stories, suggesting that collecting testimonial videos might not be the bottleneck in scaling up the video testimonial approach. Patients with UC reported the testimonials to be very relevant and helpful despite being from patients with CD, indicating that experiences from similar but not identical medical conditions may be helpful.

Limitations of this work include using a rigorous control group of patients who watch unrelated or no videos, exploring the bias introduced by only sharing testimonials from volunteers, a patient cohort more representative of the population with CD and UC, and the bias introduced by patients knowing the physicians conducting the study.

This work suggests that patient testimonials might be a low-cost intervention that can reduce health anxiety during hospital visits. As long as patients continue to be willing to share their stories, the video testimonial framework presented in this study has the potential to easily scale, especially compared to more expensive or more time-intensive interventions. Further research can help establish how anxiety reduction leads to better patient adherence to treatment plans, satisfaction with their treatment, and overall outcomes. This work also highlights the possibility that patient testimonials can be helpful in other anxiety-inducing health situations, as well as in other conditions outside of IBD.

## Appendix

Multimedia Appendix 1 Supplementary details on data.

## Abbreviations

CD: Crohn disease
HADS: Hospital Anxiety and Depression Scale
HADS-A: Hospital Anxiety and Depression Scale–Anxiety
IBD: inflammatory bowel disease
UC: ulcerative colitis
VAS-A: Visual Analog Scale for Anxiety

## Data Availability

The data sets generated during and/or analyzed during the current study are not publicly available due to patient privacy.
