# Supplementary material for: Reducing Health Anxiety in Patients With Inflammatory Bowel Disease Using Video Testimonials: Pilot Assessment of a Video Intervention
Source: JMIR Form Res. 2023 Feb 9;7:e39945. doi: 10.2196/39945 (PMC9951069; doi:10.2196/39945)
Supplement: Multimedia Appendix 1 [file formative_v7i1e39945_app1.docx]

Supplementary Materials

#### DIPEx Selected Video List

Required Viewing List:

1. 診断を受けた際の体験談 (Experience when receiving a diagnosis), https://www.dipex-j.org/movie/mp4/crohn/cd24kimoti1.mp4, 2:44
2. 診断を受けた際の体験談 (Experience when receiving a diagnosis), https://www.dipex-j.org/movie/mp4/crohn/cd16kimoti1.mp4, 1:08
3. 治療の種類：薬物療法 (Type of treatment: Drug therapy), https://www.dipex-j.org/movie/mp4/crohn/cd19kusuri1.mp4, 1:54
4. 治療の種類：生物学的製剤 (Type of treatment: Biologic), https://www.dipex-j.org/movie/mp4/crohn/cd24kusuri1.mp4, 2:18
5. 先輩患者からのメッセージ (Message from a senpai) https://www.dipex-j.org/movie/mp4/crohn/cd14message1.mp4, 1:46
6. 先輩患者からのメッセージ (Message from a senpai) https://www.dipex-j.org/movie/mp4/crohn/cd19message1.mp4, 2:14

#### Data Exclusion

One patient didn't complete a pre-video VAS-A score and was excluded from the relevant results.

Of the patients who correctly completed the VAS-A survey, 4 did not complete the HADS survey and were excluded from HADS related statistics.

One patient did not complete all questions of the usability and satisfaction surveys and was not included in the relevant results.

Some studies remove data points that are too many standard deviations from the mean. Our dataset had 4 patients who were more than 2.5 standard deviations from the mean anxiety change, which we included in the data due to our chosen test statistic being relatively robust to such outliers.
